# Large asymptomatic pheochromocytoma with incidental metastatic midgut carcinoid tumor

**DOI:** 10.1210/jcemcr/luag192

**Published:** 2026-07-22

**Authors:** Michael Salzle, Anna Szafran-Swietlik, Meredith LaRue, Jon Finan, Joshua Ziehm, Tobias Carling

**Affiliations:** Department of Endocrinology, HCA Healthcare/USF Morsani College of Medicine GME/HCA Florida Bayonet Point Hospital, Bayonet Point, FL 34667, USA; Department of Endocrinology, HCA Healthcare/USF Morsani College of Medicine GME/HCA Florida Bayonet Point Hospital, Bayonet Point, FL 34667, USA; Department of Surgery, Hospital for Endocrine Surgery HCA Florida Healthcare, Tampa, FL 33615, USA; Department of Pathology, Hospital for Endocrine Surgery HCA Florida Healthcare, Tampa, FL 33615, USA; Department of Surgery, Hospital for Endocrine Surgery HCA Florida Healthcare, Tampa, FL 33615, USA; Department of Surgery, Hospital for Endocrine Surgery HCA Florida Healthcare, Tampa, FL 33615, USA; Carling Adrenal Center, Tampa, FL 33615, USA

**Keywords:** pheochromocytoma, adrenal, adrenalectomy, midgut, carcinoid, surgery

## Abstract

A 79-year-old asymptomatic woman was referred for a large adrenal incidentaloma found on imaging for painless hematuria. Computed tomography (CT) showed a hyperenhancing mass of the left adrenal gland with central necrosis, calcification, mass effect, and measuring 9.1 × 8.3 cm. Biochemical evaluation revealed markedly elevated 24-hour urine and plasma metanephrine and normetanephrine levels, strongly suggesting pheochromocytoma. On the morning of surgery, a dedicated adrenal-protocol CT confirmed the large left pheochromocytoma and incidentally detected a previously unrecognized partially calcified soft-tissue mass in the anterior mesentery. The patient underwent uncomplicated left laparoscopic, hand-port-assisted, transabdominal adrenalectomy. Using the same hand-port incision, the mesenteric mass was removed and frozen section analysis revealed mesenteric metastasis from midgut carcinoid tumor. The primary small bowel carcinoid tumor and mesentery were then resected en bloc, followed by primary intestinal anastomosis. She recovered well and was discharged after 48 hours. Final pathology confirmed pheochromocytoma coexisting with metastatic midgut carcinoid tumor. This case highlights the rare coexistence of 2 distinct neuroendocrine tumors, underscores the importance of careful review of imaging in patients with pheochromocytoma for unanticipated findings, and demonstrates the value of intraoperative frozen section analysis combined with a tailored, minimally invasive surgical approach.

## Introduction

Pheochromocytomas are rare catecholamine-secreting tumors arising from chromaffin cells located in the adrenal medulla or along the sympathetic nervous system chain [1]. The disease can arise sporadically or be associated with inherited conditions and germline mutations. Approximately 70% of all patients with pheochromocytoma/paraganglioma can be assigned to 1 of 3 main molecular clusters with correlations to phenotypes, clinical behavior, and have previously been reviewed [2, 3]. Pheochromocytomas may manifest with episodic symptoms of anxiety, palpitations, sweating, and headaches. However, more critically, they may cause an adrenergic crisis, leading to life-threatening hypertensive emergencies, arrhythmias, heart failure, or intracranial hemorrhage. For this reason, pheochromocytomas require prompt consideration and efficient diagnosis and treatment to minimize associated morbidity and mortality [4]. Conversely, some pheochromocytomas can be “clinically silent,” characterized by the absence of signs and symptoms associated with catecholamine excess [5].

Clinically silent and nonsecretory pheochromocytomas can be challenging and are often diagnosed only after surgical and pathological inspection [6]. Like pheochromocytomas, intestinal carcinoid tumors are also rare neuroendocrine tumors (NETs) that arise from enterochromaffin cells and are located ubiquitously throughout the gastrointestinal tract. Interestingly, their incidence has increased 4-fold between 1973 and 2004 and now reaches 0.67 cases per 100 000 in the United States, likely because of increased awareness and better diagnostic strategies [7]. They can be associated with inherited syndromes, multifocal in 30% to 40% of cases, and secrete biogenic amines or hormones like serotonin, leading to symptoms including flushing, diarrhea, bronchospasm, and carcinoid heart disease. More commonly, the tumors are nonfunctioning and only discovered incidentally by endoscopy or by advanced disease stages by imaging depicting mesenteric lymph node and/or liver metastasis [8]. Diagnosing pheochromocytomas and carcinoid tumors requires a multimodal approach, including biochemical, imaging, and histopathological evaluation. We describe a patient with a large, asymptomatic pheochromocytoma with a concomitant incidentally found metastatic midgut carcinoid tumor. It emphasizes the unorthodox presentation of 2 rare neuroendocrine tumors and imperative proper investigation of incidentalomas. Finally, multidisciplinary collaboration, meticulous review of preoperative imaging for unanticipated findings, the use of intraoperative frozen section analysis, and personalized minimally invasive endocrine surgery proved beneficial in this unique case [9].

## Case presentation

A 79-year-old woman presented to the endocrinology clinic in July 2025 after being referred by her primary care physician for a left-sided adrenal incidentaloma. The adrenal mass was discovered after she underwent computed tomography (CT) imaging for painless hematuria that was resolved by the time of her initial consultation. The patient was asymptomatic; specifically, she denied episodes of palpitation, tremor, sweating, hypertension, hypotension, hyperglycemia, obstipation, flushing, diarrhea, bronchospasm, or other suggestive symptoms of a neuroendocrine lesion. The initial CT images showed a large hyperenhancing mass seen in the region of the left adrenal gland with central areas of necrosis and calcification measuring at least 8.1 cm in size. There was mass effect on all adjacent structures including the kidney inferiorly, raising initial concern for malignancy. There were no acute intra-abdominal or pelvic findings, and no evidence of lymphadenopathy. Dehydroepiandrosterone sulfate, renin, aldosterone, plasma and urine metanephrine and normetanephrine levels, and a 1-mg dexamethasone suppression test were ordered as part of routine biochemical work for an adrenal adenoma. Her medical, surgical, social, and family history were otherwise noncontributory. On physical examination, her blood pressure was well controlled without medications, and the rest of her vital signs and physical examination were unremarkable.

## Diagnostic assessment

The biochemical evaluation was consistent with pheochromocytoma. The 24-hour urine metanephrine was 582 µg/24 hours (SI: 2.95 µmol/24 hours) (reference range 36-209 µg/24 hours [SI: 0.18—1.06 µmol/24 hours]) and normetanephrine 14 834 µg/24 hour (SI: 75 µmol/24 hours) (reference range 131-612 µg/24 hours [SI: 0.72-3.34 µmol/24 hours]) and a plasma metanephrine of 181 pg/mL (SI: 917 pmol/L) (reference range 0-88 pg/mL [SI: 0-808 pmol/L]) and normetanephrine 6962.5 pg/mL (SI: 38 000 pmol/L; reference range 0-285 pg/mL [SI: 0-900 pmol/L]). Additional biochemical assessment included a normal cortisol level after a 1-mg dexamethasone suppression test at 1.7 µg/dL (SI: 46.9 nmol/L) (reference range: <1.8 µg/dL [SI: <49.7 nmol/L]), aldosterone < 1 ng/dL (SI: <27.7 pmol/L) (reference range: 0-30 ng/dL [SI: 0-832 pmol/L]), renin 0.289 ng/mL (SI: 370.5 pmol/L) (reference range: 0.167-5.380 ng/mL [SI: 128− 4149 pmol/L]), dehydroepiandrosterone sulfate 40.5 µg/dL (SI 1.405 µmol/L) (reference range 13.9-142.8 µg/dL [SI: 1.7-10.3 µmol/L]), and testosterone <3 ng/dL (SI: <0.10 nmol/L) (reference range 3.0-67 ng/dL [SI: 0.4-1.2 nmol/L]).

On the morning of surgery for operative planning, a dedicated adrenal-protocol CT confirmed the large heterogenous hypervascular mass centered in the position of the left adrenal gland with prominent peripheral soft-tissue components and possibly central necrotic component, measuring 9.1 × 8.3 × 6.3 cm and contained an internal coarse calcification (Fig. 1A-1C). In addition, there was a partially calcified soft-tissue mass within the anterior mesentery below the level of the umbilicus measuring 2.2 cm, suspicious for a metastasis in the context of the adrenal findings (Fig. 1D-1F), which had not been demonstrated on previous cross-sectional imaging. Of note, all CT phases performed used contrast enhancement and thus, no calculations of unenhanced Hounsfield unit attenuation values was not performed.

**Figure 1:**
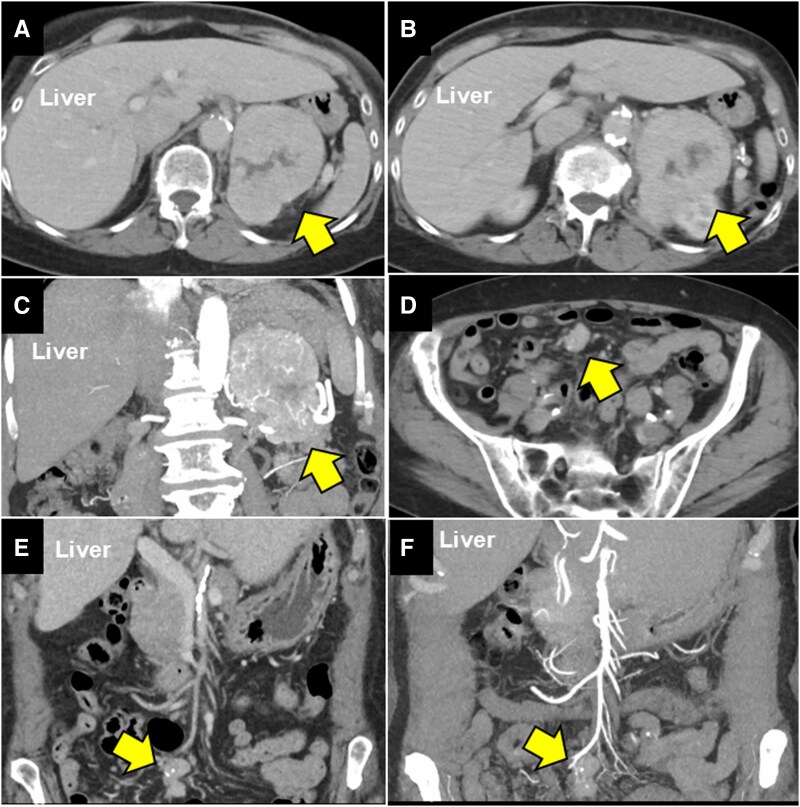
Preoperative imaging demonstrating a large left pheochromocytoma with an incidental anterior mesenteric mass. (A,B) Axial CT images (venous phase) showing a 9.1 × 8.3 × 6.3 cm large heterogenous hypervascular mass arising from the left adrenal gland with prominent soft-tissue components, and possibly central necrotic components (arrows). (C) Coronal CT image (arterial phase) of the same mass depicting significant hypervascularity of the mass (arrow). (D) Axial CT image (venous phase) demonstrating an incidental partially calcified soft-tissue mass within the anterior mesentery measuring 2.2 cm (arrow). (E,F) The same 2.2-cm mesenteric mass (arrows) is demonstrated on coronal CT images in venous and arterial phases, respectively.

## Treatment

Preoperatively, the patient was initially placed on doxazosin 1 mg daily, then gradually titrated to 2 mg twice per day before surgery, without difficulty, and was advised to increase her salt intake to 3 to 5 g per day. Although the mini back scope adrenalectomy (aka posterior retroperitoneoscopic approach) is the preferred operation for almost all patients with pheochromocytoma [9], given the size of the tumor, and the need to address the mesenteric lesion, a transabdominal approach was selected. Thus, the patient underwent a left laparoscopic, hand-port-assisted transabdominal adrenalectomy without complication. Using the same hand-port incision, the mesenteric mass was removed, and frozen section analysis revealed mesenteric metastasis from a midgut NET. The primary small bowel NET and mesentery were then resected, en bloc, followed by intestinal primary anastomosis. Intraoperatively, there was no hemodynamic instability, significant tachycardia, arrhythmia, or bleeding. On gross pathology, the left adrenal mass consisted of a 209.9-gram, 9.7 × 7.5 × 6.0 cm intact adrenal gland lesion (Fig. 2A, white arrow). The mesenteric mass consisted of a yellow-red lesion measuring 4.5 × 3.2 × 2.0 cm (Fig. 2A, yellow arrow). The small bowel resection consisted of a portion of small bowel that measured 25.0 cm long × 2.0 cm in diameter. The patient tolerated the procedure well, and no intensive care admission was required. Given the larger transabdominal incision, and intestinal anastomosis, the patient spent 48 hours in the hospital before discharge for pain management and assessment of bowel function (as opposed to the customary <23 hours after mini back scope adrenalectomy). A standardized, institutional cosyntropin stimulation test, performed the morning after surgery, demonstrated a robust cortisol response (cortisol = 18.5 µg/dL; 510.4 nmol/L, at 60 minutes after administration of 250 mcg cosyntropin), ruling out postadrenalectomy hypocortisolism [5].

**Figure 2:**
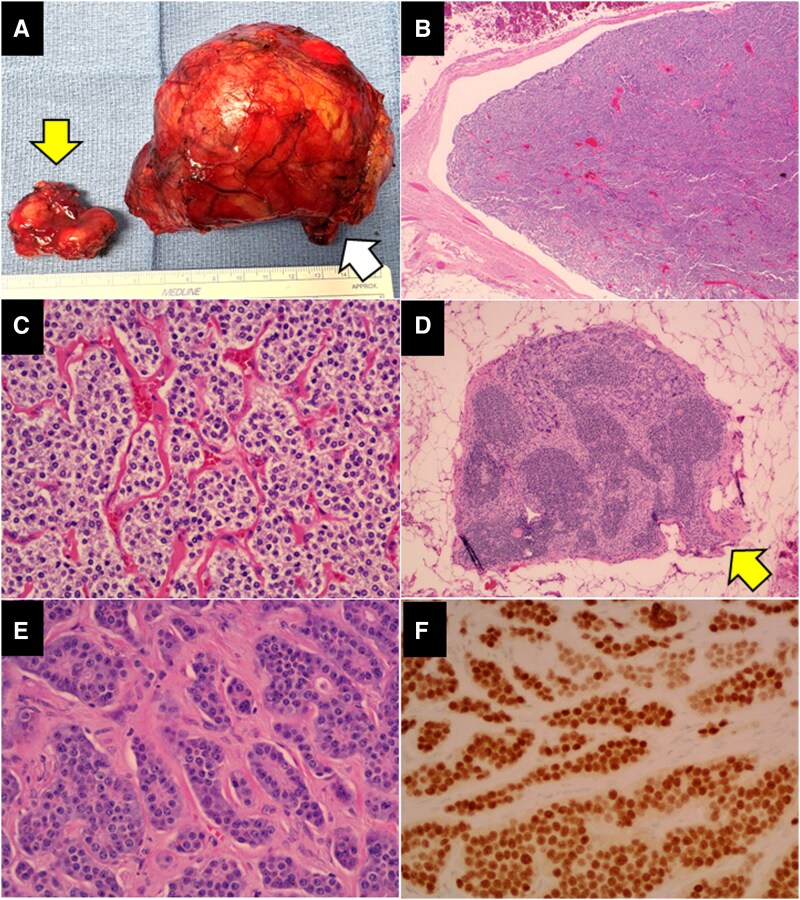
Gross and histopathology from a case with a large left pheochromocytoma and midgut carcinoid tumor metastatic to the anterior mesentery. Representative images from surgical pathology diagnosing a large left pheochromocytoma and midgut carcinoid tumor metastatic to the anterior mesentery. (A) Gross pathology demonstrating the large left pheochromocytoma (white arrow), and the anterior mesenteric mass (yellow arrow), both resected en bloc. (B-F) Histopathology using hematoxylin-eosin (HE; B-E) as well as CDX-2 immunostaining (F), using magnification of 20× (B), 40× (D), and 200× (C, E, F), with the pheochromocytoma displaying vascular invasion (B), and typical features of classical “zellballen” pattern; cluster of pale eosinophilic chromaffin cells surrounded by vascularized septa (C). (D) Mesenteric mass surrounded by adipose tissue (arrow) proven to be metastatic from a midgut carcinoid with typical histological features (ie, uniform cells with finely granular nuclear chromatin that are arranged in a trabecular-like fashion) (E) and characteristic immunoreactivity using CDX-2 antibodies (F).

## Outcome and follow-up

The histopathological analysis confirmed the diagnosis of pheochromocytoma (Fig. 2) showing a low Ki-67 index of 2% and positive immunostaining for chromogranin, GATA3 and S100. The mesenteric mass proved to be metastatic from a midgut NET displaying Ki-67 less than 2% and pankeratin, CDX2, and chromogranin immunopositivity. The primary bowel lesion consisted of a multifocal (4 distinct lesions, ranging in size from 0.4 to 1.5 cm) well-differentiated neuroendocrine tumor, and 4 of 6 resected mesenteric lymph nodes were positive for metastatic disease. The primary bowel lesions and the metastatic mesenteric lesions with lymph nodes had the exact same histological and immunostaining pattern. Collectively, the findings support a pheochromocytoma completely distinct from a midgut NET that metastasized to the mesenteric lymph node compartment. Postoperatively, the patient remained asymptomatic, and plasma metanephrine and normetanephrine normalized to 3.2 pg/mL (SI: 0.18 nmol/L) and 115.7 pg/mL (SI: 0.63 nmol/L), respectively. Her 5-hydroxyindoleacetic acid was normal at 2.9 mg/24 hours (SI 15.2 µmol/24 hours) (reference range 0-14.9 mg/24 hours [SI: 10-47 µmol/24 hours]). The chromogranin A was minimally elevated at 169.4 ng/mL (SI 169.4 µg/L) (reference range 0-101.8 ng/mL [SI: 0-102 µg/L]). She established care with an oncologist who ordered a Gallium-68 DOTA-0-Tyr^3^-octreotate scan to evaluate metastatic/residual disease. Imaging showed a 1.2 × 0.9 cm nodule in the superior mediastinum anterior to the trachea with mild increased tracer uptake (SUV 2.2). There was no other abnormal tracer uptake in the mediastinum. There was increased uptake within the small bowel loop in the right pelvis below anastomosis level (SUV 6.9) but no other abnormality on localization CT. Neither of these findings were thought to be clinically significant and related to her neuroendocrine tumors, and thus her oncologist recommended surveillance with repeat biochemical evaluation, and possibly future imaging to assess for recurrence. The patient was encouraged and referred to genetic counselling and testing for identifying a putative susceptibility pathogenic variant of her disease but declined.

## Discussion

Despite the initial impression of an uncomplicated case of a large asymptomatic pheochromocytoma, preoperative imaging and surgical exploration revealed a more convoluted case after discovering an incidental small bowel and mesenteric mass proven to be a midgut NET with metastasis. The coexistence of a pheochromocytoma and a carcinoid tumor is exceedingly rare, with only a handful of cases ever reported in literature [10]. There are no well-established pathogenic germline variants definitively linked to the rare cooccurrence of midgut NET and pheochromocytoma in the same patients, and unfortunately the patients declined genetic testing. Although there are data lacking on long-term outcomes of a simultaneous pheochromocytoma and metastatic carcinoid tumor due to the rarity of these comorbidities, isolated small intestinal NETs have a median overall survival of about 8.4 years [11].

The patient lacked classical pheochromocytoma symptoms, categorizing this as a clinically silent pheochromocytoma. Silent pheochromocytomas are relatively rare, occurring only 8% of the time [12]. However, a recent study from a high-volume adrenal surgery hospital demonstrated that silent pheochromocytomas may comprise as much as 15% of surgically removed pheochromocytomas and tend to be smaller, with an average size of 2.9 cm [6]. There tends to be a positive correlation between a patient's catecholamine levels and the presence of symptoms [13]. Therefore, the discrepancy between the patient's asymptomatic presentation and her markedly elevated catecholamine levels and robust size (9.7 cm) of the adrenal mass was rather anomalous. This case also underscored the need for a multidisciplinary approach in evaluation and management of a pheochromocytomas and carcinoid tumor with metastasis for optimal outcomes.

Lifelong biochemical monitoring and imaging are critical to detecting recurrence of both pheochromocytoma with a rate of at least 10%, as well as midgut NETs [14]. In the current patient, plasma metanephrine, normetanephrine, and chromogranin levels will likely serve as excellent biochemical tumor markers to detect a possible recurrence of pheochromocytoma and carcinoid tumors, respectively. However, given the patient's complete surgical removal (R0 resection) of both the pheochromocytoma and midgut NET, absence of aggressive features (eg, low Ki-67 index) on pathology, and complete biochemical remission based on postoperative laboratory values and functional imaging, the prognosis appears excellent. Personalized, minimally invasive surgery in expert hands remains the definitive mainstay of treatment for most pheochromocytomas, and when feasible, also for midgut NETs to reduce risks of intraoperative and postoperative complications [9].

## Learning points

- The simultaneous occurrence of a pheochromocytoma and carcinoid tumor is exceedingly rare and has been reported only sporadically in the literature.
- The case underscores the importance of careful review of imaging in patients with pheochromocytoma for unanticipated findings, such as concomitant neuroendocrine intestinal tumors.
- Tailored, minimally invasive surgical approaches can be used with intraoperative frozen section analysis to effectively diagnose and cure both pheochromocytoma and metastatic midgut carcinoid tumors.

## Contributors

All authors made individual contributions to authorship. M.S., A.S.S., M.L., and T.C. were responsible for data collection, analysis, diagnosis, and management of the patient. T.C. performed the operation, with assistance from J.Z., and they obtained intraoperative and gross pathology images. J.F. provided pathology expertise and prepared the histology images. M.S. and T.C. wrote the initial draft. All authors were involved in manuscript preparation and reviewed and approved the final draft.

## Data Availability

Data sharing is not applicable to this article as no datasets were generated or analyzed during the current study.
